# Development and Internal Validation of the BRICS Score: A Preoperative Clinical and Hematological Model for Predicting High-Grade and Muscle-Invasive Bladder Cancer

**DOI:** 10.3390/cancers18203282

**Published:** 2026-10-12

**Authors:** Raul-Dumitru Gherasim, Daniel Porav-Hodade, Călin Chibelean, Septimiu Voidazan, Ciprian Todea-Moga, Oliver-Arpad Vida, Veronica-Maria Ghirca, Rareș-Florin Vascul, Tibor Lorand Reman, Orsolya Katalin Ilona Martha

**Affiliations:** 1 Department of Urology, George Emil Palade University of Medicine, Pharmacy, Science, and Technology of Târgu Mureș, 540139 Târgu Mureș, Romania; raul-dumitru.gherasim@umfst.ro (R.-D.G.); ciprian.todea@umfst.ro (C.T.-M.); arpad.vida@umfst.ro (O.-A.V.); maria.ghirca@umfst.ro (V.-M.G.); raresvascul@gmail.com (R.-F.V.); tibor.reman@umfst.ro (T.L.R.); orsolya.martha@umfst.ro (O.K.I.M.); 2 Department of Urology, Târgu Mureș Clinical Hospital, P-ța Bernády György, Nr. 6, 540072 Târgu-Mureș, Romania; 3 Department of Epidemiology, George Emil Palade University of Medicine, Pharmacy, Science, and Technology of Târgu Mureș, 540139 Târgu Mureș, Romania; septimiu.voidazan@umfst.ro

**Keywords:** bladder cancer, muscle-invasive bladder cancer, high-grade bladder cancer, NLR, systemic inflammation, prediction model, risk stratification, BRICS

## Abstract

Background/Objectives: We developed and internally validated the Bladder Risk Index using Clinical and Systemic biomarkers (BRICS), a preoperative prediction model designed to estimate the likelihood of high-grade (HG) disease and muscle-invasive bladder cancer (MIBC) in patients undergoing their first transurethral resection of bladder tumor (TURBT). Methods: This retrospective single-center study included 161 patients with newly diagnosed urothelial bladder cancer. The BRICS-HG model included tumor size, neutrophil-to-lymphocyte ratio (NLR), and creatinine-to-hemoglobin ratio (CHR), while the BRICS-MIBC model was based on tumor size and NLR. Model performance was assessed in terms of discrimination, calibration, and Brier score, together with exploratory decision curve analysis. Internal validation was performed using 2000 bootstrap resamples. Results: MIBC was identified in 20 patients (12.4%). BRICS-HG demonstrated limited discriminatory ability, with an apparent AUC of 0.649 and an optimism-corrected AUC of 0.623 (95% bootstrap interval: 0.541–0.703). BRICS-MIBC achieved moderate discrimination, with an apparent AUC of 0.735 and an optimism-corrected AUC of 0.709 (95% bootstrap interval: 0.588–0.826). Across the bootstrap resamples, the model combining tumor size and NLR was selected in 53.9% of cases, reflecting uncertainty in predictor selection. Conclusions: BRICS-HG should be regarded as an exploratory model, and its current performance does not support clinical application. BRICS-MIBC demonstrated moderate discrimination after internal validation; however, its performance should be interpreted cautiously given the small number of MIBC events. Validation in larger, independent cohorts, ideally across multiple centers, is necessary before the model can be considered for clinical implementation.

## Simple Summary

Bladder cancer grade and muscle invasion are usually confirmed after transurethral resection and histopathological examination. Estimating the risk of aggressive disease before surgery could provide useful information for patient assessment and treatment planning. In this study, we developed and internally validated two prediction models, collectively referred to as BRICS. The model for predicting MIBC showed more promising predictive performance than the model for predicting high-grade disease. To facilitate risk estimation, both models were implemented in a freely accessible web-based calculator. BRICS represents a simple approach that does not require advanced imaging analysis or specialized infrastructure. However, validation in larger, independent patient populations is required before the models and calculator can be recommended for clinical use.

## 1. Introduction

Bladder cancer (BC) is a heterogeneous disease in which prognostic and therapeutic management depend substantially on tumoral grade and stage. The distinction between non-muscle invasive bladder cancer (NMIBC) and muscle invasive (MIBC) is essential, because invasion of the muscularis propria substantially influences the therapeutic strategy and subsequent oncological management [1]. Similarly, the distinction between low-grade (LG) or high-grade (HG) disease has major implications in progression and recurrence risk and subsequent treatment [2]. Therefore, early assessment of tumor aggressiveness may provide clinically relevant information for patient counseling and management planning [3].

However, the definitive determination of the tumor’s grade and stage relies on the histopathological examination following transurethral resection of bladder tumor (TURBT). Before TURBT, assessment of tumor aggressiveness must therefore rely on preoperative clinical information in the absence of definitive histopathological findings. [4]. At this stage, certain preoperative characteristics may suggest aggressive tumor behavior and may contribute to estimating the probability of HG or MIBC disease [5].

Systemic inflammation has been implicated in cancer development and progression, increasing the interest in readily available blood-derived inflammatory biomarkers. The neutrophil-to-lymphocyte ratio (NLR), lymphocyte-to-monocyte ratio (LMR) and platelet-to-lymphocyte ratio (PLR) have been investigated in patients with bladder cancer and are associated with various oncological outcomes [6]. More recently, the creatinine-to-hemoglobin ratio (CHR) has been proposed as a potential marker for urothelial carcinomas [7]. Unlike advanced imaging-based or molecular approaches, these laboratory parameters are inexpensive, routinely available, and can be obtained before TURBT, without requiring specialized infrastructure.

Given the biological heterogeneity of BC, a single biomarker is unlikely to capture the full spectrum of tumor aggressiveness. Integrating hematological biomarkers with clinical and imaging data could provide a more comprehensive assessment of individual risk. Such approaches may improve the identification of patients at risk of aggressive disease without requiring definitive histopathological findings or complex molecular analyses [8,9,10].

Based on the available information, the objective of our study was to develop and internally validate a clinical and hematological prediction model for the preoperative risk stratification of patients with newly diagnosed bladder tumor. The model framework was termed BRICS (Bladder Risk Index using Clinical and Systemic biomarkers). By integrating clinical and tumor characteristics with biomarkers derived from routine laboratory tests, BRICS aims to estimate the preoperative probability of HG disease and MIBC (≥pT2).

## 2. Materials and Methods

### 2.1. Study Design and Population

Patients with newly diagnosed (“de novo”) bladder tumors were included in the study. All included patients were newly diagnosed with BC and underwent their first TURBT in our department. Patients with a prior history of BC or other non-urothelial malignancies were excluded. Additionally, we excluded all the patients with systemic inflammatory or hematological disorders that could potentially affect blood parameters. Additional exclusion criteria included other active malignancies and insufficient clinical or laboratory data.

Individual predicted probabilities of HG and MIBC were calculated from the final multivariable logistic regression models using the corresponding linear predictors and logistic transformation. The final BRICS-HG and BRICS-MIBC equations were subsequently implemented in a web-based calculator to facilitate individualized preoperative risk estimation. The calculator automatically derives NLR and CHR from the entered laboratory parameters and returns the estimated probabilities of HG and MIBC. Given the absence of external validation, the calculator was developed as a research and educational tool and was not intended to replace standard diagnostic assessment or histopathological evaluation.

The study was conducted in accordance with the Declaration of Helsinki and was approved by our Institutional Ethics Committee (no. 13351/2026).

### 2.2. Preoperative Variables

Histopathological evaluation of the index TURBT specimen provided the reference standard for tumor grade and pathological stage. To minimize inter-observer variability, all pathology slides were reviewed by a single dedicated uropathologist specializing in bladder oncology. Tumors were graded according to the 2016 WHO classification system. Muscle-invasive bladder cancer (MIBC) was defined as pathologically documented invasion of the muscularis propria (≥pT2), whereas non-muscle-invasive bladder cancer (NMIBC) encompassed lesions lacking detrusor invasion. Because muscularis propria was identified in every initial TURBT specimen, pathological staging was based on specimens containing the relevant anatomical layer. The MIBC endpoint used for model development was restricted to finding from the initial TURBT; data from repeat TURBTs or subsequent radical surgeries were not incorporated into the endpoint definition. Accordingly, separate prediction models were developed for MIBC and HG disease.

Tumor size was assessed endoscopically and defined as the maximum diameter, in millimeters, of the largest tumor. Size was estimated by the same surgical team using a standardized approach, with a 26-Ch resectoscope and resection loops of known dimensions (KARL STORZ SE & Co. KG, Tuttlingen, Germany) used as visual references.In patients with multifocal disease, the largest tumor diameter was recorded, rather than the sum of all tumor diameters, while multifocality was recorded separately as a binary variable. The tumor size used for model development was based solely on endoscopic assessment and was not derived from or adjusted according to preoperative imaging.

### 2.3. Pathological Outcomes

Histopathological evaluation of the initial TURBT specimen served as the reference standard for the tumor grade and pathological stage. The primary outcome was MIBC, defined as ≥pT2 disease, whereas the secondary outcome was HG. Separate prediction models were developed for the two outcomes. Tumor grade was assessed according to the 2016 WHO classification and categorized as low-grade or high-grade. The corresponding G1–G3 grade, when reported by the pathologist, was recorded as additional pathological information but was not used to define the study endpoints.

### 2.4. Statistical Analysis

Continuous variables were expressed as mean ± standard deviation or median (IQR), as appropriate. Normality was assessed using the Shapiro–Wilk test. Non-normally distributed variables were compared using the Mann–Whitney U test, while categorical variables were analyzed using Pearson’s chi-square or Fisher’s exact test, as appropriate. Clinical and laboratory characteristics were compared according to tumor grade and MIBC status.

Candidate predictors were selected based on their pre-TURBT availability, clinical plausibility and established associations with aggressive bladder cancer phenotypes. While univariable logistic regression was used to describe individual associations with each outcome, it was not applied as a strict significance threshold for multivariable inclusion. To prevent predictor redundancy, creatinine was omitted when CHR was present. To address distributional skewness, hematological ratios were log_2_-transformed—allowing ORs to represent a two-fold increase, whereas tumor size was analyzed continuously per 10 mm increment. For BRICS-HG, 11 parsimonious candidate models were evaluated, including tumor size alone and tumor size combined with one or two hematological markers (NLR, PLR, LMR, and CHR). Candidate models compared in terms of model fit (AIC), discriminative ability, prediction error and structural parsimony. Based on its balanced performance across these criteria, the model incorporating tumor size, NLR, and CHR was selected as the final specification.

To reduce the risk of overfitting given the limited number of MIBC events, candidate BRICS-MIBC models were restricted to a maximum of two predictors. Five parsimonious candidate models were evaluated: tumor size alone and tumor size paired individually with NLR, PLR, LMR, or CHR. Model comparison was based on the Akaike Information Criterion (AIC), discriminative performance, prediction error, and model parsimony. For internal validation of BRICS-MIBC, the complete model-selection workflow was repeated across 2000 bootstrap resamples. Within each iteration, all five candidate models were refitted, and the model with the lowest AIC was selected. The selected model was evaluated in both the bootstrap sample and the original dataset to estimate performance optimism arising from model fitting and selection. Selection frequency for each candidate model was also recorded across all resamples. Given the limited number of MIBC events, a sensitivity analysis using Firth’s penalized logistic regression was performed for the final BRICS-MIBC model. The same predictors and transformations were retained (tumor size per 10 mm increase and NLR per two-fold increase) to allow direct comparison with the conventional logistic regression model. This analysis was used to assess the sensitivity of coefficient estimates to potential small-sample bias and did not alter the specification of the primary prediction model. Uncertainty around the optimism-corrected AUC and Brier score was quantified from the empirical bootstrap distributions. For each of the 2000 bootstrap resamples, optimism-corrected performance was derived from the corresponding estimate of optimism, and 95% bootstrap intervals were defined by the 2.5th and 97.5th percentiles of the resulting distributions.

Potential departures from linearity in the logit for continuous variables in the final models were evaluated using restricted cubic splines. In the BRICS-MIBC model, no significant evidence of nonlinearity was observed for either tumor size (*p* = 0.609) or log_2_-transformed NLR (*p* = 0.918). Similarly, in the BRICS-HG model, no significant evidence of nonlinearity was observed for tumor size (*p* = 0.134) or CHR (*p* = 0.794); however, a potential nonlinear pattern was detected for NLR (*p* = 0.039). Given the exploratory nature of this finding and the added complexity of spline terms, the linear specification was retained.

There were no missing data for the variables included in the analyses; therefore, no imputation was performed, no observations were excluded because of missing data, and all analyses were conducted using the complete cohort (*n* = 161).

Decision curve analysis (DCA) was conducted to evaluate the potential net benefit of using predicted HG or MIBC risk to inform intensified preoperative evaluation and surgical planning, rather than to guide definitive oncological management. Threshold probabilities were interpreted as the minimum predicted risk at which intensified preoperative assessment would be considered clinically justified, reflecting the trade-off between false-positive and false-negative classifications. In the absence of established probability cut-offs for BRICS-guided preoperative planning, thresholds were examined across a continuum and framed as exploratory rather than fixed clinical decision boundaries. Net benefit was compared with the default strategies of intensified assessment for all patients or for none. To account for optimism in the BRICS-MIBC decision curves, net benefit optimism was quantified across 2000 bootstrap resamples, with the complete five-model selection workflow repeated within each iteration. For each threshold probability, optimism was defined as the difference between the bootstrap net benefit and the net benefit obtained by applying the bootstrap-selected model to the original cohort; mean optimism was subsequently subtracted from the apparent net benefit of the final BRICS-MIBC model. Consequently, the optimism-corrected decision curve reflects uncertainty arising from both model estimation and candidate-model selection. For BRICS-HG, optimism correction was performed by refitting the final model without repeating a variable-selection procedure. The final prediction equations were derived from the regression coefficients estimated in the complete development cohort. For BRICS-HG, the linear predictor was:

LP(HG) = 0.851450 + 0.184166 × (tumor size [mm]/10) + 0.289953 × log_2_(NLR) + 0.338290 × log_2_(CHR).

For BRICS-MIBC, the linear predictor was:

LP(MIBC) = −4.086810 + 0.561342 × (tumor size [mm]/10) + 0.422921 × log_2_(NLR).

For both models, the individual predicted probability was calculated as P = 1/[1 + exp(−LP)]. NLR was defined as the absolute neutrophil count divided by the absolute lymphocyte count, whereas CHR was defined as serum creatinine (mg/dL) divided by hemoglobin (g/dL).

Model discrimination was assessed using receiver operating characteristic (ROC) curves and the area under the curve (AUC), with 95% confidence intervals. Statistical analyses were performed using R statistical software (**version 4.5.1;** R Foundation for Statistical Computing, Vienna, Austria), with statistical significance set at *p* < 0.05. Model development and reporting followed TRIPOD recommendations [11].

## 3. Results

### 3.1. Study Population and Baseline Characteristics

**Figure 1:**
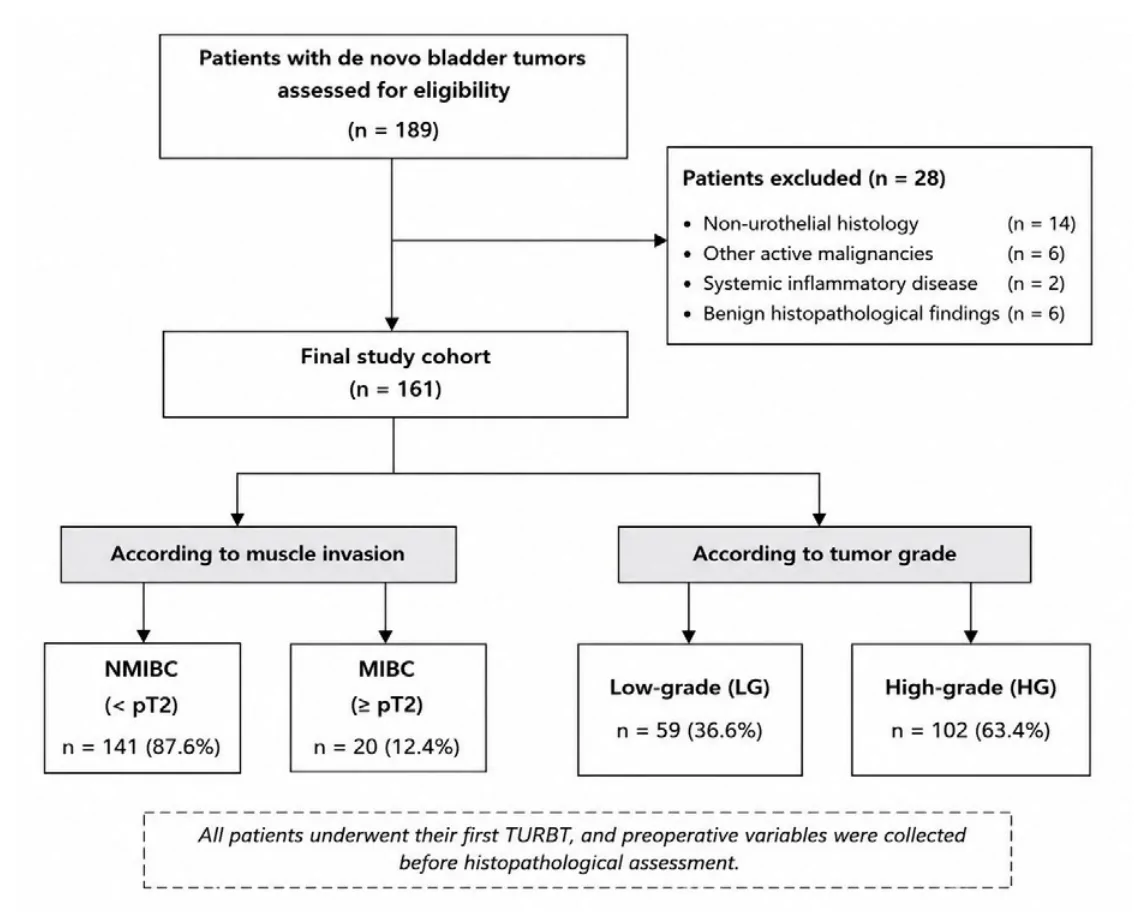
Flowchart of patient selection.

Of the 189 patients initially assessed for eligibility, 28 were excluded: 14 due to non-urothelial histology, six due to other active malignancies, two due to systemic inflammatory disease and six because of benign histopathological findings. The final study cohort comprised 161 patients who underwent their first TURBT. Muscularis propria was present in the initial TURBT specimen in all 161 patients (100%). Based on histopathological assessment of the initial TURBT specimens, 141 patients (87.6%) were classified as having NMIBC and 20 (12.4%) as having MIBC (≥pT2). Regarding tumor grade, 59 patients (36.6%) had LG disease and 102 (63.4%) had HG disease. Patient selection is illustrated in Figure 1.

### 3.2. Comparison of Preoperative Characteristics by Tumor Grade

Among the 161 patients, 59 (36.6%) had LG and 102 (63.4%) had HG disease. Patients with HG disease had significantly higher preoperative creatinine levels (*p* = 0.023), NLR (*p* = 0.013), PLR (*p* = 0.025), LMR (*p* = 0.003), and CHR (*p* = 0.030), whereas LMR was significantly lower (*p* = 0.003). No significant differences were observed in age, sex, smoking status, hematuria, hypertension, tumor size, or multifocality. Continuous variables are presented as median (IQR). Detailed results are provided in Table 1.

### 3.3. Preoperative Characteristics According to Muscle Invasion

The NMIBC group comprised141 (87.6%) patients, whereas the MIBC group comprised 20 patients (12.4%). Patients with MIBC had significantly larger tumors than those with NMIBC (median 30 vs. 15 mm, with *p* = 0.002) and significantly higher preoperative creatinine levels (1.28 vs. 1.02 mg/dL, *p* = 0.019). No significant differences were observed in age, sex, smoking status, hematuria, hypertension, multifocality or PLR. Patients with MIBC had higher CHR (0.109 vs. 0.084, *p* = 0.009) while LMR was significantly lower (2.07 vs. 3.06, *p* = 0.041). NLR tended to be higher in the MIBC group, although the difference did not reach statistical significance (4.48 vs. 3.31, *p* = 0.072). The complete results are presented in Table 2.

### 3.4. Predictors of High-Grade Disease

Univariable logistic regression was used to evaluate associations between preoperative clinical, tumor-related, and laboratory variables and HG disease. Higher creatinine levels, NLR, and CHR were significantly associated with increased odds of HG disease. A two-fold increase in creatinine was associated with an OR of 1.92 (95% CI: 1.11–3.31; *p* = 0.020), while a two-fold increase in NLR and CHR were associated with ORs of 1.46 (95% CI: 1.08–1.97; *p* = 0.013) and 1.60 (95% CI: 1.05–2.44; *p* = 0.029), respectively. Conversely, higher LMR was associated with lower odds of high-grade disease (OR: 0.72 per two-fold increase; 95% CI: 0.54–0.97; *p* = 0.028). Tumor size was not significantly associated with HG disease (OR: 1.26 per 10 mm increase; 95% CI 0.97–1.63; *p* = 0.084). The remaining clinical parameters and PLR were not significantly associated with HG disease in our univariable analysis (Table 3).

Candidate multivariable models were subsequently evaluated to identify a parsimonious combination of preoperative predictors for HG disease. To minimize predictor redundancy, creatinine was not simultaneously included with CHR, while alternative models incorporating either NLR or LMR were compared. Across the 11 candidate models evaluated, the specification incorporating tumor size, NLR, and CHR provided the most favorable balance among model fit, discriminative performance, prediction error, and parsimony (Supplementary Table S1). Therefore, this model was retained as the BRICS-HG model. In the multivariable model, the adjusted ORs were 1.20 (95% CI: 0.92–1.56; *p* = 0.169) per 10 mm increase in tumor size, 1.34 (95% CI: 0.98–1.82; *p* = 0.067) per two-fold increase in NLR, and 1.40 (95% CI: 0.91–2.16; *p* = 0.126) per two-fold increase in CHR (Table 4). None of the individual predictors retained statistical significance after mutual adjustment. Model performance and internal validation are reported in Section 3.6.

### 3.5. Predictors of Muscle-Invasive Disease

Univariable logistic regression was used to assess the associations between preoperative clinical, tumor-related and laboratory variables and the presence of MIBC. Tumor size and NLR were significantly associated with increased odds of MIBC. Each 10 mm increase in tumor size was associated with an OR of 1.77 (95% CI: 1.27–2.47; *p* < 0.001), while a two-fold increase in NLR was associated with an OR of 1.59 (95% CI 1.09–2.22; *p* = 0.017). Hematuria and CHR were associated with increased odds of MIBC, although these associations did not reach statistical significance. The remaining variables were not significantly associated with muscle-invasive disease in our univariable analysis.

We subsequently tested five parsimonious candidate models for MIBC prediction: tumor size as single variable, and tumor size paired individually with NLR, PLR, LMR or CHR. To reduce the risk of overfitting given the limited number of MIBC events, model complexity was restricted to a maximum of two predictors. Within the development cohort, the model combining tumor size and NLR achieved the lowest AIC (110.433) and Brier score (0.093) among all candidate models, while demonstrating moderate discrimination (AUC = 0.735) (Supplementary Table S1). Consequently, this specification was selected as the final BRICS-MIBC model. In the multivariable analysis, tumor size remained significantly associated with MIBC, with an adjusted OR of 1.75 per 10 mm increase (95% CI: 1.24–2.48; *p* = 0.002). Similarly, a two-fold increase in NLR was independently associated with increased odds of MIBC (adjusted OR: 1.53; 95% CI: 1.03–2.26; *p* = 0.035) (Table 5). Model performance and internal validation are reported in Section 3.6.

In the sensitivity analysis, the final BRICS-MIBC model was refitted using Firth’s penalized logistic regression. The resulting coefficient estimates were similar to those obtained with conventional logistic regression. For tumor size, the OR was 1.72 per 10 mm increase (95% CI: 1.23–2.42), compared with 1.75 (95% CI: 1.24–2.48) in the primary analysis. For NLR, the OR was 1.48 per two-fold increase (95% CI: 1.01–2.18), compared with 1.53 (95% CI: 1.03–2.26) in the primary analysis. Overall, Firth’s penalization resulted in only minor changes in the effect estimates, without materially altering the direction or magnitude of the associations (Supplementary Table S2).

### 3.6. Performance and Internal Validation of the BRICS Models

The final BRICS models were selected by comparing candidate multivariable models based on model fit, discrimination, calibration, prediction error, and parsimony. For HG disease, the combination of tumor size, NLR and CHR provided the most favorable overall balance across these criteria and was retained as the BRICS-HG model. For MIBC prediction, the combination of tumor size and NLR provided the most favorable balance of model fit, discrimination, prediction error, and parsimony and was retained as the BRICS-MIBC model. Given the limited number of MIBC events, model complexity was restricted to two predictors to reduce the risk of overfitting (Table 6).

**Figure 2:**
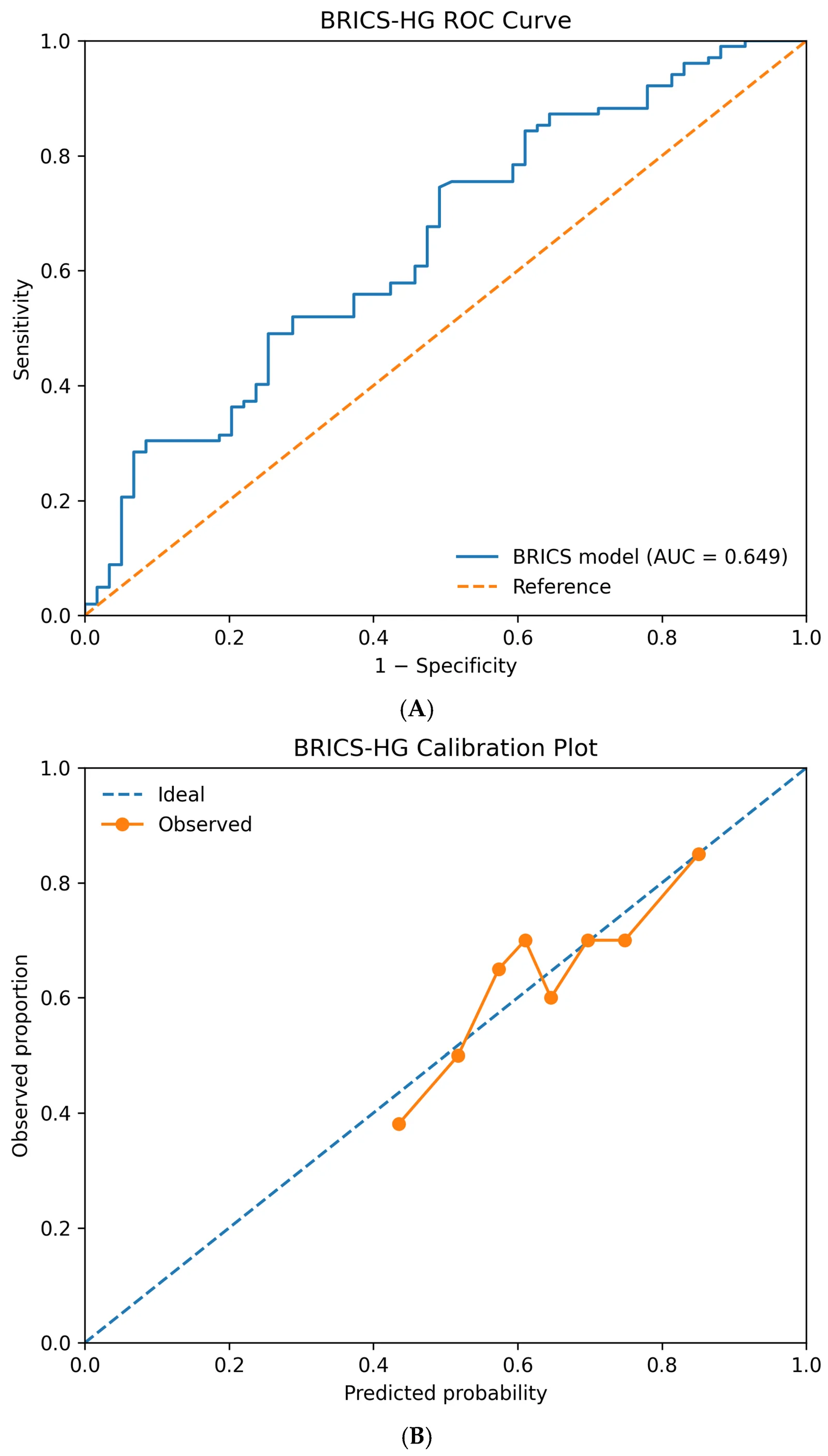
(**A**) BRICS-HG ROC curve. (**B**) BRICS-HG calibration plot.

The BRICS-HG model showed limited discrimination for HG disease, with an apparent AUC of 0.649 (95% CI: 0.556–0.735). Following internal validation with 2000 bootstrap resamples, the estimated optimism was 0.026, resulting in an optimism-corrected AUC of 0.623 (95% bootstrap interval: 0.541–0.703) (Figure 2A).

**Figure 3:**
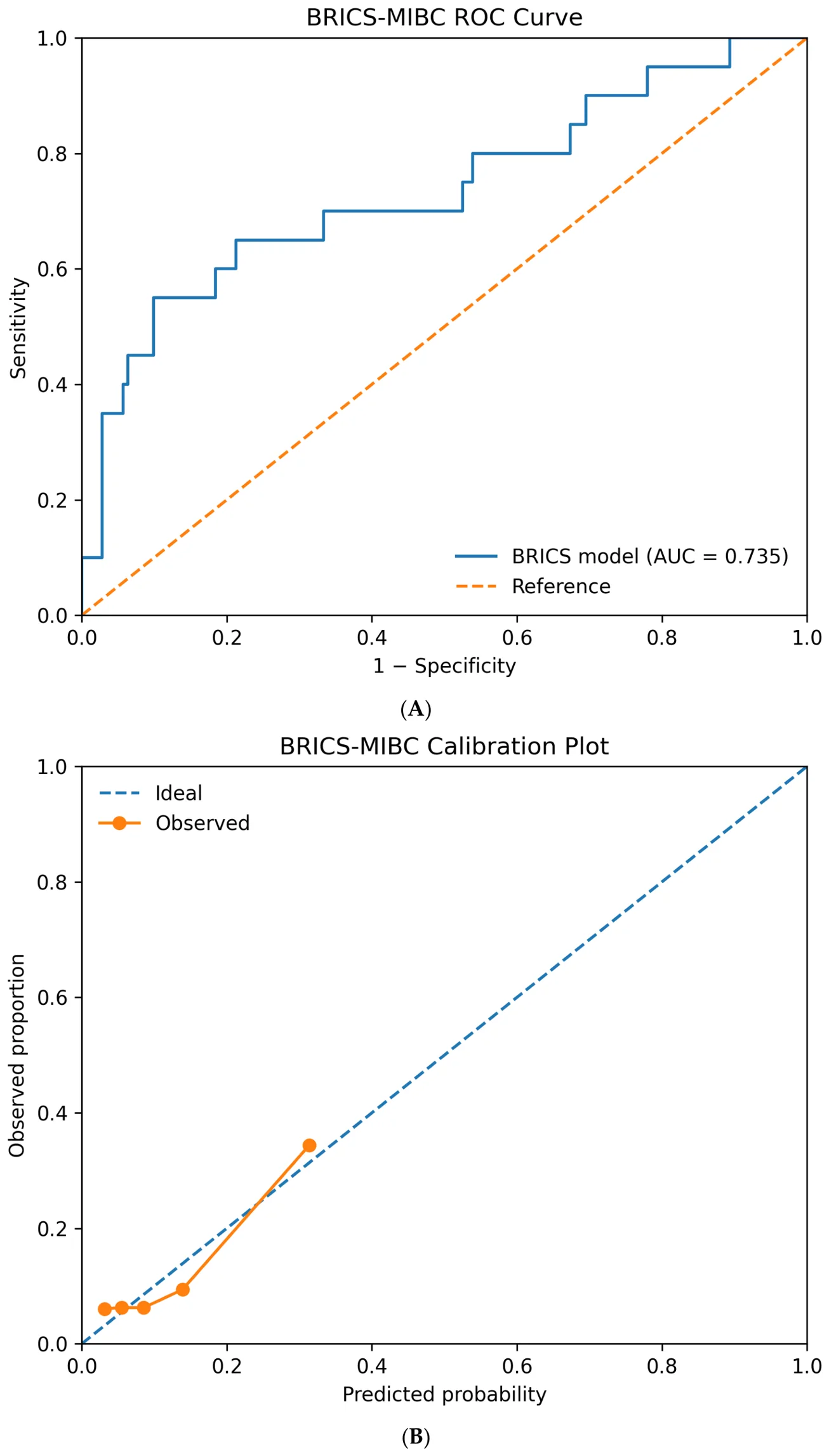
(**A**) BRICS-MIBC ROC curve. (**B**) BRICS-MIBC calibration plot.

The apparent Brier score was 0.216 and the optimism-corrected Brier score was 0.226 (95% bootstrap interval: 0.205–0.255). The median bootstrap calibration slope was 0.813, while the median calibration intercept was 0.088 (Figure 3A). These findings indicate limited discriminatory ability and some degree of optimism in model performance.

The BRICS-MIBC model showed moderate discrimination, with an apparent AUC of 0.735 (95% CI: 0.591–0.864). After repeating the complete candidate-model selection procedure within 2000 bootstrap resamples, the estimated optimism was 0.026, resulting in an optimism-corrected AUC of 0.709 (95% bootstrap interval: 0.588–0.826). The apparent and optimism-corrected Brier scores were 0.093 and 0.100, respectively, with a 95% bootstrap interval of 0.070–0.138 for the corrected estimate. The median bootstrap calibration slope was 0.853 and the median calibration intercept was −0.235. The tumor size plus NLR model was selected in 1077 of 2000 bootstrap resamples (53.9%), indicating substantial uncertainty in predictor selection (Figure 2B and Figure 3B).

The point estimate of internally validated discrimination was higher for BRICS-MIBC than for BRICS-HG (optimism-corrected AUC: 0.709 vs. 0.623); however, considerable uncertainty remained around BRICS-MIBC performance because of the limited number of MIBC events and variability in predictor selection. These findings should therefore not be interpreted as evidence of model stability or robustness.

### 3.7. Exploratory Decision Curve Analysis and Risk Estimation of the BRICS Models

Decision curve analysis was performed as an exploratory assessment of whether model-guided intensified preoperative assessment and planning could provide greater net benefit than strategies of intensified assessment for all patients or for no patients. Threshold probabilities represent the minimum predicted risk at which intensified assessment would be considered justified and should not be interpreted as validated clinical cut-offs.

For BRICS-MIBC, the decision evaluated by DCA was not whether to perform TURBT, which remains the standard diagnostic procedure for suspected bladder tumors, but whether a higher predicted risk of MIBC might warrant more intensive preoperative assessment and planning. This may include additional or expedited staging, prioritization of the diagnostic and therapeutic pathway, planning TURBT with the possibility of invasive disease in mind, earlier multidisciplinary or specialist referral, and more detailed patient counseling. Accordingly, the “intensified-assessment-for-all” and “intensified-assessment-for-none” strategies refer to applying these additional measures to all patients or to none, respectively.

**Figure 4:**
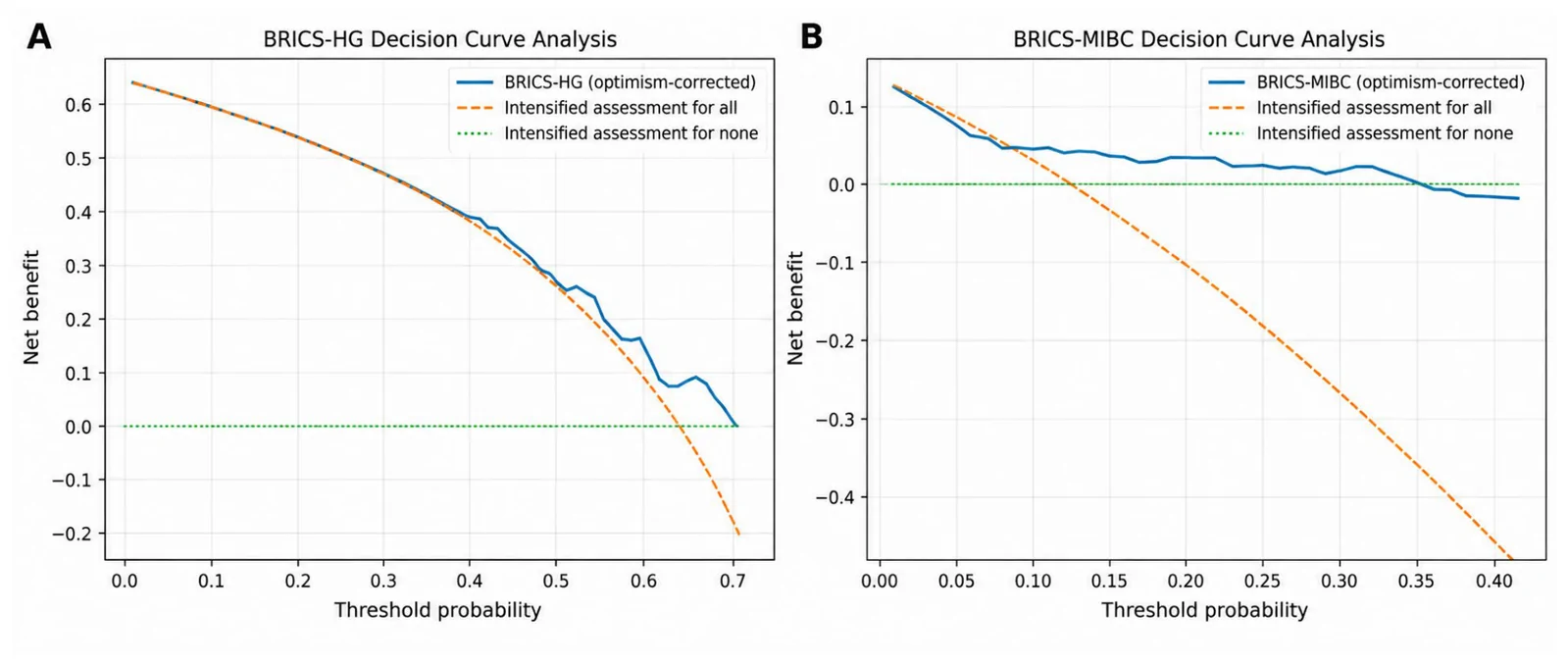
Decision curve analysis of the BRICS models. (**A**) BRICS-HG and (**B**) BRICS-MIBC. Net benefit is shown across threshold probabilities representing the minimum predicted risk at which intensified preoperative assessment and planning would be considered justified. The reference strategies represent intensified assessment for all patients and intensified assessment for no patients. Threshold probabilities are exploratory and do not represent validated clinical cut-offs. The BRICS-MIBC curve was corrected for optimism using 2000 bootstrap resamples, with the complete five-model candidate selection procedure repeated within each resample. The BRICS-HG curve was corrected for optimism by bootstrap refitting of the final model without repeating model selection.

For BRICS-HG, DCA was corrected for optimism by bootstrap refitting of the final model. Because model selection was not repeated within the HG bootstrap procedure, the corresponding curve should be interpreted as fixed-model optimism-corrected rather than fully selection-adjusted. The model showed a modest net benefit compared with both reference strategies, with the main range of potential benefit observed at threshold probabilities of approximately 0.39–0.70. At a threshold probability of 0.60, the optimism-corrected net benefit was 0.129, compared with 0.084 for intensified assessment for all patients and 0 for intensified assessment for no patients. These findings should be interpreted as exploratory and do not establish the clinical utility of BRICS-HG (Figure 4A).

For BRICS-MIBC, after optimism correction incorporating the complete candidate-model selection procedure within 2000 bootstrap resamples, the model showed greater net benefit than both reference strategies across an approximate threshold-probability range of 0.09–0.34. At threshold probabilities of 0.10, 0.15, and 0.20, the optimism-corrected net benefits were 0.042, 0.033, and 0.031, respectively. These thresholds are presented for illustration rather than as recommended clinical cut-offs. Given the limited number of MIBC events, these findings should be regarded as exploratory (Figure 4B).

In addition to their classification performance, the final logistic models allow estimation of individualized probabilities of HG disease and MIBC using routinely available preoperative parameters. BRICS-HG incorporates tumor size, NLR, and CHR, whereas BRICS-MIBC uses tumor size and NLR. The models are intended to complement, rather than replace, standard diagnostic assessment and histopathological evaluation following TURBT. Pending external validation, their potential role is to support preoperative risk stratification and decisions regarding the intensity of preoperative assessment and planning, rather than to determine whether TURBT should be performed or to replace histopathological diagnosis.

To facilitate individualized risk estimation, the final BRICS-HG and BRICS-MIBC models were implemented as a freely accessible web-based calculator. Based on the entered preoperative tumor and laboratory parameters, the calculator automatically derives NLR and CHR and provides predicted probabilities of HG disease and MIBC. The BRICS Preoperative Risk Calculator is publicly available online and is intended for research and educational purposes pending external validation. The BRICS Preoperative Risk Calculator is publicly available at https://raulgherasimgrd-ops.github.io/BRICS-FINAL-SCORE/ (accessed on 3 October 2026).

## 4. Discussion

In the present study, we developed and internally validated two preoperative models for estimating the probability of high-grade (HG) and muscle-invasive bladder cancer (MBIC) using clinical, tumor-related and hematological parameters, which are routinely available before TURBT. BRICS-HG, which incorporated tumor size, NLR, and CHR, showed limited discrimination after internal validation, with an optimism-corrected AUC of 0.623 and a calibration slope of 0.813. In comparison, BRICS-MIBC, which incorporated tumor size and NLR, showed moderate discrimination, with an apparent AUC of 0.735 and an optimism-corrected AUC of 0.709 after accounting for model-selection uncertainty. The median bootstrap calibration slope was 0.853. However, the limited number of MIBC events and variability in predictor selection indicate that these internally validated performance estimates remain uncertain and should not be interpreted as evidence of model stability or robustness. Taken together, these results suggest that routinely available preoperative clinical, tumor-related, and laboratory parameters may contribute to the early estimation of tumor aggressiveness, particularly the risk of muscle-invasive disease. The BRICS models should therefore be viewed as tools for preoperative risk stratification, providing additional information before definitive histopathological results, rather than as replacements for TURBT or pathological diagnosis. These findings are consistent with the growing evidence supporting the feasibility of preoperative risk estimation in bladder cancer, although many contemporary prediction models rely on advanced imaging, radiomics or machine-learning approaches.

Our findings are consistent with those reported by Xiong et al. [12], who developed a preoperative model for predicting unfavorable pathological features in patients with an initial diagnosis of BC. Their clinical model identified tumor size and NLR as independent predictors and achieved an AUC of 0.742 in the testing cohort. BRICS-MIBC retained the same combination of preoperative variables and achieved an apparent AUC of 0.735 and an optimism-corrected AUC of 0.709. Although direct comparison of model performance is not appropriate because of differences in outcome definitions and validation strategies, the identification of tumor size and NLR as predictors in both studies supports their potential relevance for the preoperative assessment of aggressive BC. Xiong et al. further demonstrated that incorporating CT-based radiomic features improved predictive performance beyond the clinical model. In contrast, BRICS was intentionally developed using routinely available parameters, with an emphasis on accessibility across different clinical settings.

Li et al. [13] developed an MRI-based radiomics nomogram for the preoperative prediction of histological grade in NMIBC and reported an AUC of 0.931 for the combined radiomics-clinical model, compared with 0.745 for the clinical model alone. Rather than aiming to outperform imaging-based models, BRICS was developed with an emphasis on simplicity, which may facilitate individualized risk estimation in clinical settings, where sophisticated imaging-based prediction tools are not available.

Tumor size was an important tumor-related variable associated with muscle-invasive disease in our cohort. Each 10 mm increase in tumor size was associated with a 75% increase in the adjusted odds of MIBC, whereas its association with high-grade disease was not statistically significant after multivariable analysis. The relevance of tumor size in the preoperative assessment of bladder cancer aggressiveness has also been reported in recent studies. Deng et al. [14] found tumor size to be independently associated with pathological grade, while NLR was also associated with tumor grade in univariate analysis. Similarly, Kang et al. [15] incorporated a tumor size threshold of <3 cm into a CT-based prediction model for identifying patients with a high probability of NMIBC. Despite differences in study populations, outcomes, and modeling strategies, these findings support the potential relevance of tumor size in the assessment of bladder cancer aggressiveness. In BRICS, tumor size was not considered in isolation, but was integrated with markers of systemic inflammation to provide a simple preoperative risk-estimation model before definitive histopathological assessment.

Systemic inflammation has emerged as a relevant aspect of bladder cancer biology, with several inflammatory indices investigated as potential markers of tumor aggressiveness and prognosis. In our cohort, a higher inflammatory burden was generally associated with more aggressive disease: patients with HG disease had higher NLR, PLR and CHR with a lower LMR. Patients with MIBC had lower LMR and higher CHR, while NLR also higher, although the difference did not reach statistical significance. These findings are consistent with recent evidence supporting an association between systemic inflammation and adverse clinicopathological features in BC. In a meta-analysis including 20 studies and 5,594 patients, Zhang et al. [16] reported that elevated PLR was significantly associated with tumor stage ≥ T2 (OR 1.92, 95% CI 1.24–2.97), as well as poorer overall and recurrence-free survival. Importantly, not all inflammatory indices retained independent associations after multivariable adjustment in our cohort. NLR was retained in both BRICS models and remained independently associated with MIBC, while PLR and LMR were not included in the final models. These findings suggest that inflammatory biomarkers may be more informative when combined with clinical and tumor-related characteristics rather than used alone. Although inflammatory and hematological markers have been associated with aggressive bladder cancer in previous studies, statistical association does not necessarily translate into sufficient individual-level discrimination for clinical prediction, as illustrated by the performance of BRICS-HG.

Clinical utility and calibration provide complementary information regarding the reliability and potential clinical relevance of individual risk. In our cohort, BRICS-MIBC yielded an apparent AUC of 0.735 and an optimism-corrected AUC of 0.709 after accounting for the complete candidate-model selection workflow during bootstrap validation. The median bootstrap calibration slope was 0.853. These more conservative estimates emphasize the importance of accounting for model-selection uncertainty, particularly given the limited number of MIBC events BRICS-HG showed limited predictive performance. Its apparent AUC of 0.649 decreased to an optimism-corrected AUC of 0.623 (95% bootstrap interval: 0.541–0.703), with a median bootstrap calibration slope of 0.813. This level of discrimination is insufficient for use as a stand-alone clinical prediction tool and suggests that tumor size, NLR, and CHR capture only a limited portion of the information relevant to HG disease. Accordingly, BRICS-HG should be regarded as an exploratory model requiring further development rather than as a clinically applicable prediction tool. The findings of Jaromin et al. [17] also support the potential role of routinely available biomarkers in the preoperative identification of muscle-invasive disease. They evaluated SII, SIRI, PIV and PLR derived from pre-TURBT blood tests. Their multivariable models achieved AUC values ranging from 0.795 to 0.821 for the prediction of MIBC, further supporting the potential value of these biomarkers before the definitive pathological result.

Beyond discrimination, recent biomarker-based models have also emphasized the importance of calibration and clinical utility. In a large multicenter study, Feng et al. [18] developed an inflammation-based nomogram incorporating NLR and PLR together with clinicopathological variables and evaluated its performance in terms of discrimination, calibration, and decision curve analysis. Although their model addressed overall survival after radical cystectomy rather than preoperative prediction of muscle invasion, their approach illustrates the importance of evaluating prediction models across multiple performance domains. The DCA findings should be interpreted as exploratory rather than as evidence of established clinical utility. For BRICS-MIBC, the selection-adjusted analysis suggested a positive net benefit compared with both reference strategies across threshold probabilities of approximately 0.09–0.34. However, these thresholds are not validated clinical cut-offs, and no single threshold can be recommended from the present retrospective dataset. Rather, the threshold probability represents the minimum predicted risk at which the consequences and resource requirements of intensified preoperative assessment would be considered acceptable in relation to the risk of missing MIBC. Establishing an actionable threshold would require external validation and prospective evaluation incorporating clinical consequences, resource use, and patient and clinician preferences. From a clinical perspective, the potential value of BRICS lies primarily in its reliance on parameters routinely available before TURBT and its ability to translate these variables into individualized probabilities of HG and MIBC through a simple web-based calculator. More complex imaging-based approaches, such as the model developed by Wei et al. [19], have demonstrated higher predictive performance. In contrast to these more resource-intensive imaging-based models, BRICS was designed to provide a simpler and more accessible approach based on routinely available clinical and laboratory parameters. The calculator should be regarded as an accessible tool for preoperative risk stratification rather than as an alternative to histopathological diagnosis or more sophisticated imaging-based models. External validation is nevertheless required before the calculator can be recommended for clinical use.

Several strengths of the present study should be acknowledged. The BRICS models were developed exclusively using preoperative variables routinely available before TURBT, and the study population was restricted to patients with newly diagnosed bladder tumors, undergoing their first resection. An additional methodological strength was the uniformity of the pathological reference standard: all initial TURBTs were performed by the same surgical team, muscularis propria was present in all specimens, and all cases were evaluated by the same dedicated uropathologist. Model performance was evaluated beyond discrimination by incorporating calibration, prediction error, decision curve analysis and internal validation using 2000 bootstrap resamples.

Nevertheless, several limitations should be considered. First, this was a single-center study with a relatively small sample size, which may limit the generalizability of our findings [20]. This limitation holds particular weight for BRICS-MIBC, given that only 20 patients presented with muscle-invasive disease, constraining the available information for model development and introducing uncertainty into predictor selection and performance metrics. Despite limiting model complexity, repeating the full candidate-model selection procedure across 2000 bootstrap resamples and using Firth’s penalized logistic regression yielded similar coefficient estimates. However, these approaches cannot fully address the limitations associated with the small number of MIBC events (n = 20). The resulting uncertainty in predictor selection and performance estimation should therefore be considered when interpreting BRICS-MIBC. Internal validation can quantify optimism and model-development uncertainty but cannot establish model stability, transportability, or performance in independent patient populations or clinical settings. Finally, BRICS-HG should be regarded as an exploratory model with limited discrimination. The decrease in performance after bootstrap correction indicates some degree of optimism in the development cohort, while the optimism-corrected AUC remains insufficient for stand-alone clinical prediction. Further model development may require additional predictors carrying information not captured by routine clinical and hematological variables, followed by validation in an independent cohort. Accordingly, the current BRICS-HG model should not be used to guide clinical management.

Although muscularis propria was present in all initial TURBT specimens and all cases were assessed by the same dedicated uropathologist, the MIBC endpoint was based on the pathological stage assigned at the initial TURBT. Subsequent pathological findings from repeat TURBT or definitive surgery were not systematically incorporated into the model-development endpoint. Therefore, the present study does not assess longitudinal stage concordance or the frequency of subsequent pathological upstaging.

Further studies should focus on external validation [21] of both BRICS models in larger independent patient populations. Ideally, validation should be performed prospectively across multiple centers to assess the reproducibility, calibration and clinical utility. For BRICS-MIBC, the internally validated estimate indicated moderate discrimination; however, its performance should be interpreted cautiously given the limited number of MIBC events (n = 20). External validation in larger, preferably multicenter cohorts with more MIBC events is therefore needed to assess model performance and generalizability across different clinical populations.

External validation may also identify the need for model recalibration or updating before clinical implementation. The current web-based calculator should therefore be regarded as a research and educational tool at this stage, with its coefficients and risk estimates updated, if necessary, according to the results of future external validation. Ultimately, prospective multicenter evaluation will be necessary to determine whether BRICS can provide clinically meaningful support for preoperative risk stratification in patients with newly diagnosed bladder tumors.

## 5. Conclusions

The BRICS models investigated whether routinely available clinical and hematological variables could contribute to preoperative estimation of HG and MIBC risk before the first TURBT. BRICS-HG showed limited discrimination after internal validation and should be considered exploratory; its current performance does not support clinical use, and further model development is required. BRICS-MIBC showed moderate discrimination after internal validation, but these performance estimates remain uncertain because of the limited number of MIBC events and variability in predictor selection. Independent external validation in larger, preferably multicenter cohorts is required before either model can be considered for clinical implementation.

## Figures and Tables

**Table 1:** Preoperative characteristics according to tumor grade.

Variable	LG (n = 59), Median (IQR)	HG (n = 102), Median (IQR)	*p*-Value
Age, years	66.0 (59.5–78.0)	69.5 (63.0–74.8)	0.694
Tumor size, mm	15 (5–20)	15 (5.3–30)	0.144
Creatinine, mg/dL	0.98 (0.80–1.22)	1.15 (0.88–1.44)	0.023
NLR	2.83 (1.70–5.32)	3.77 (2.54–6.78)	0.013
PLR	129.88 (89.44–193.57)	170.59 (109.95–248.73)	0.025
LMR	3.84 (2.46–5.39)	2.56 (1.73–3.90)	0.003
CHR	0.08 (0.06–0.11)	0.09 (0.07–0.13)	0.030

**Table 2:** Preoperative characteristics according to muscle invasion. (Continuous variables are presented as median (IQR) and were compared using the Mann–Whitney U test. Categorical variables are presented as n (%) and were compared using Pearson’s chi-square test or Fisher’s exact test, as appropriate.)

Variable	NMIBC (n = 141)	MIBC (n = 20)	*p*-Value
Age, years	68.0 (61.0–76.0)	67.5 (63.0–73.0)	0.770
Sex, n (%)			0.956
Male	108 (76.6)	16 (80.0)	
Female	33 (23.4)	4 (20.0)	
Smoking status, n (%)			0.248
Never smoker	47 (33.3)	10 (50.0)	
Former smoker	21 (14.9)	1 (5.0)	
Current smoker	73 (51.8)	9 (45.0)	
Hematuria, n (%)			0.144
No	56 (39.7)	4 (20.0)	
Yes	85 (60.3)	16 (80.0)	
Hypertension, n (%)			1.000
No	40 (28.4)	6 (30.0)	
Yes	101 (71.6)	14 (70.0)	
Tumor size, mm	15 (5–20)	30 (15–40)	0.002
Multifocality, n (%)			0.321
Solitary	57 (40.4)	11 (55.0)	
Multiple	84 (59.6)	9 (45.0)	
Creatinine, mg/dL	1.02 (0.81–1.34)	1.28 (1.08–1.71)	0.019
NLR	3.31 (2.01–5.80)	4.48 (2.88–9.11)	0.072
PLR	135.90 (96.75–226.14)	181.46 (127.58–215.92)	0.347
LMR	3.06 (1.97–4.90)	2.07 (1.46–3.44)	0.041
CHR	0.084 (0.061–0.117)	0.109 (0.093–0.183)	0.009

**Table 3:** Univariable logistic regression analysis of preoperative predictors of high-grade bladder cancer.

Predictor	Univariable OR (95% CI)	*p*-Value
Age, per 10-year increase	1.08 (0.80–1.45)	0.618
Female vs. male	0.70 (0.33–1.47)	0.344
Former vs. never smoker	1.27 (0.46–3.51)	0.642
Current vs. never smoker	1.48 (0.74–2.98)	0.270
Hematuria	1.41 (0.73–2.72)	0.309
Hypertension	1.70 (0.85–3.42)	0.135
Tumor size, per 10 mm increase	1.26 (0.97–1.63)	0.084
Multifocality	1.40 (0.73–2.67)	0.308
Creatinine, per doubling	1.92 (1.11–3.31)	0.020
NLR, per doubling	1.46 (1.08–1.97)	0.013
PLR, per doubling	1.26 (0.98–1.61)	0.074
LMR, per doubling	0.72 (0.54–0.97)	0.028
CHR, per doubling	1.60 (1.05–2.44)	0.029

**Table 4:** Univariable and multivariable logistic regression analyses of preoperative predictors of high-grade bladder cancer. (Odds ratios (ORs) are presented with 95% confidence intervals (CIs). The multivariable BRICS-HG model included tumor size, NLR, and CHR. For biomarkers with skewed distributions, ORs are expressed per two-fold increase following log_2_ transformation. Tumor size is expressed per 10 mm increase and age per 10-year increase. Variables not included in the final multivariable model are indicated by an em dash (—). A two-sided *p*-value < 0.05 was considered statistically significant.)

Predictor	Univariable OR (95% CI)	*p*-Value	Multivariable OR (95% CI)	*p*-Value
Age, per 10-year increase	1.08 (0.80–1.45)	0.618	—	—
Female vs. male	0.70 (0.33–1.47)	0.344	—	—
Former vs. never smoker	1.27 (0.46–3.51)	0.642	—	—
Current vs. never smoker	1.48 (0.74–2.98)	0.270	—	—
Hematuria	1.41 (0.73–2.72)	0.309	—	—
Hypertension	1.70 (0.85–3.42)	0.135	—	—
Tumor size, per 10 mm increase	1.26 (0.97–1.63)	0.084	1.20 (0.92–1.56)	0.169
Multifocality	1.40 (0.73–2.67)	0.308	—	—
Creatinine, per doubling	1.92 (1.11–3.31)	0.020	—	—
NLR, per doubling	1.46 (1.08–1.97)	0.013	1.34 (0.98–1.82)	0.067
PLR, per doubling	1.26 (0.98–1.61)	0.074	—	—
LMR, per doubling	0.72 (0.54–0.97)	0.028	—	—
CHR, per doubling	1.60 (1.05–2.44)	0.029	1.40 (0.91–2.16)	0.126

**Table 5:** Univariable and multivariable logistic regression analyses of preoperative predictors of muscle-invasive bladder cancer. (Odds ratios (ORs) are presented with 95% confidence intervals (CIs). The multivariable BRICS-MIBC model included tumor size and NLR. NLR was log_2_-transformed, and its OR is therefore expressed per two-fold increase. Tumor size is expressed per 10 mm increase and age per 10-year increase. Variables not included in the final multivariable model are indicated by an em dash (—). A two-sided *p*-value < 0.05 was considered statistically significant.)

Predictor	Univariable OR (95% CI)	*p*-Value	Multivariable OR (95% CI)	*p*-Value
Age, per 10-year increase	0.99 (0.64–1.53)	0.968	—	—
Female vs. male	0.82 (0.26–2.62)	0.735	—	—
Former vs. never smoker	0.22 (0.03–1.86)	0.166	—	—
Current vs. never smoker	0.58 (0.22–1.53)	0.271	—	—
Hematuria	2.64 (0.84–8.29)	0.098	—	—
Hypertension	0.92 (0.33–2.57)	0.880	—	—
Tumor size, per 10 mm increase	1.77 (1.27–2.47)	<0.001	1.75 (1.24–2.48)	0.002
Multifocality	0.56 (0.22–1.43)	0.221	—	—
Creatinine, per doubling	1.57 (0.90–2.75)	0.114	—	—
NLR, per doubling	1.59 (1.09–2.33)	0.017	1.53 (1.03–2.26)	0.035
PLR, per doubling	1.06 (0.74–1.54)	0.739	—	—
LMR, per doubling	0.68 (0.43–1.09)	0.108	—	—
CHR, per doubling	1.46 (0.93–2.29)	0.097	—	—

**Table 6:** Discrimination, calibration, and internal validation of the BRICS-HG and BRICS-MIBC models. For BRICS-MIBC, the complete candidate-model selection procedure was repeated within each of 2000 bootstrap resamples to account for optimism arising from both model fitting and model selection.

Performance Measure	BRICS-HG	BRICS-MIBC
Predictors	Tumor size + NLR + CHR	Tumor size + NLR
Apparent AUC	0.649	0.735
95% CI for apparent AUC	0.556–0.735	0.591–0.864
Bootstrap optimism	0.026	0.026
Optimism-corrected AUC	0.623	0.709
95% bootstrap interval for optimism-corrected AUC	0.541–0.703	0.588–0.826
Apparent Brier score	0.216	0.093
Optimism-corrected Brier score	0.226	0.100
95% bootstrap interval for optimism-corrected Brier Score	0.205–0.255	0.070–0.138
Bootstrap calibration slope, median	0.813	0.853
Bootstrap calibration intercept, median	0.088	−0.235
Internal validation	2000 bootstrap resamples	2000 bootstrap resamples with candidate-model selection repeated within each resample

## Data Availability

The data that support the findings of this study are available from the corresponding author upon reasonable request. The data are not publicly available due to patient privacy and ethical restrictions. To facilitate individualized risk estimation and research use of the BRICS models, a freely accessible web-based calculator was developed using the final model coefficients. The calculator allows users to input the required preoperative variables and provides the corresponding estimated probability of high-grade and muscle-invasive bladder cancer. The online calculator is available at: https://raulgherasimgrd-ops.github.io/BRICS-FINAL-SCORE/ (accessed on 3 October 2026).

## Supplementary Materials

The following supporting information can be downloaded at https://www.mdpi.com/article/10.3390/cancers18203282/s1, Table S1: Performance characteristics of candidate models evaluated during BRICS model development; Table S2. Sensitivity analysis of the BRICS-MIBC model using Firth penalized logistic regression.

## Abbreviations

The following abbreviations are used in this manuscript:

BC	Bladder Cancer
NMIBC	Non-Muscle-Invasive Bladder Cancer
MIBC	Muscle-Invasive Bladder Cancer
LG	Low-Grade
HG	High-Grade
TURBT	Transurethral Resection of Bladder Tumor
NLR	Neutrophil-to-Lymphocyte Ratio
LMR	Lymphocyte-to-Monocyte Ratio
CHR	Creatinine-to-Hemoglobin Ratio
BRICS	Bladder Risk Index using Clinical and Systemic biomarkers
UCS	Urethro-cystoscopy
PLR	Platelet-to-Lymphocyte Ratio
WHO	World Health Organization
ROC	Receiver Operating Characteristic
AUC	Area Under the Curve
CI	Confidence Interval
TRIPOD	Transparent reporting of a Multivariable Prediction Model for Individual Prognosis or Diagnosis
IQR	Interquartile Range
OR	Odds Ratio
CT	Computed Tomography
MRI	Magnetic Resonance Imaging
SII	Systemic Inflammation Response Index
PIV	Pan-Immune-Inflammation Value
SIRI	Systemic Inflammation Response Index
