# Supplementary material for: Development and Internal Validation of the BRICS Score: A Preoperative Clinical and Hematological Model for Predicting High-Grade and Muscle-Invasive Bladder Cancer
Source: Cancers (Basel). 2026 Oct 12;18(20):3282. doi: 10.3390/cancers18203282 (PMC13702414; doi:10.3390/cancers18203282)
Supplement: Supplementary file 1 [file cancers-18-03282-s001.zip › cancers-4569031-supplementary.pdf]

Supplementary Table S1. Performance characteristics of candidate models evaluated during BRICS model development

Candidate models were compared using Akaike information criterion (AIC), apparent area under the receiver operating characteristic curve (AUC), Brier score, and parsimony. Final model selection was based on the overall balance of these criteria rather than maximization of AUC alone.

A. Candidate models for high-grade disease (BRICS-HG)

Candidate model	AIC	Apparent AUC	Brier score
Tumor size	212.425	0.568	0.228
Tumor size + NLR	208.577	0.628	0.219
Tumor size + PLR	211.499	0.621	0.224
Tumor size + LMR	210.175	0.628	0.222
Tumor size + CHR	209.606	0.631	0.221
Tumor size + NLR + PLR	210.432	0.626	0.219
Tumor size + NLR + LMR	210.372	0.631	0.219
**Tumor size + NLR + CHR (final BRICS-HG)**	**208.019**	**0.649**	**0.216**
Tumor size + PLR + LMR	212.119	0.628	0.222
Tumor size + PLR + CHR	209.731	0.649	0.219
Tumor size + LMR + CHR	209.482	0.655	0.218

B. Candidate models for muscle-invasive bladder cancer (BRICS-MIBC)

Candidate model	AIC	Apparent AUC	Brier score
Tumor size	113.250	0.712	0.100
**Tumor size + NLR (final BRICS-MIBC)**	**110.433**	**0.735**	**0.093**
Tumor size + PLR	115.236	0.727	0.100
Tumor size + LMR	113.190	0.736	0.096
Tumor size + CHR	113.223	0.744	0.099

Notes: Lower AIC and Brier scores indicate better model fit and lower prediction error, respectively, whereas higher AUC indicates better discrimination. The selected final models are shown in bold. For BRICS-MIBC, model complexity was deliberately restricted because only 20 MIBC events were available. Abbreviations: AIC, Akaike information criterion; AUC, area under the receiver operating characteristic curve; CHR, creatinine-to-hemoglobin ratio; LMR, lymphocyte-to-monocyte ratio; NLR, neutrophil-to-lymphocyte ratio; PLR, platelet-to-lymphocyte ratio.

Supplementary Table S2. Sensitivity analysis of the BRICS-MIBC model using Firth penalized logistic regression

The primary maximum-likelihood logistic regression model and the Firth penalized logistic regression sensitivity analysis used the same predictor specification: tumor size per 10-mm increase and NLR per two-fold increase.

Predictor	Maximum-likelihood β	Maximum-likelihood OR (95% CI)	Firth β	Firth OR (95% CI)
Intercept	−4.0868	—	−3.9339	—
Tumor size, per 10 mm	0.5613	1.75 (1.24–2.48)	0.5452	1.72 (1.23–2.42)
NLR, per two-fold increase	0.4229	1.53 (1.03–2.26)	0.3946	1.48 (1.01–2.18)

Abbreviations: β, regression coefficient; CI, confidence interval; NLR, neutrophil-to-lymphocyte ratio; OR, odds ratio. NLR was log_2-transformed; therefore, its OR represents the change in odds associated with a two-fold increase in NLR. Firth regression was performed as a sensitivity analysis and did not replace the primary BRICS-MIBC model.
